# Multidimensional characterization of allergic rhinitis in Mysuru, South India: A cluster-based approach

**DOI:** 10.1016/j.jacig.2026.100664

**Published:** 2026-02-11

**Authors:** Padukudru Anand Mahesh, Attahalli Shivanarayanprasad Praveena, Mohammed Kaleem Ullah, Mandya Venkateshmurthy Greeshma, Jayaraj Biligere Siddaiah, Jefferson Daniel, Devasahayam Jesudas Christopher, Krishnarajah Nirantharakumar, Adel H. Mansur, Glenis Scadding, David C. Wraith, Mamidipudi Thirumala Krishna

**Affiliations:** a Department of Respiratory Medicine, JSS Medical College, JSS Academy of Higher Education & Research, Mysuru, India; b Department of Studies in Statistics, University of Mysore, Mysuru, India; c Department of Biotechnology and Bioinformatics, JSS Academy of Higher Education and Research, Mysuru, India; d Center of Excellence in Molecular Biology and Regenerative Medicine Laboratory (DST-FIST Supported Center and ICMR Collaborating Center of Excellence), Department of Biochemistry (DST-FIST Supported Department), JSS Medical College, JSS Academy of Higher Education & Research, Mysuru, India; e Department of Pulmonology, Christian Medical College, Vellore, India; f Department of Applied Health Research, School of Health Sciences, University of Birmingham, Birmingham, United Kingdom; g Department of Respiratory Medicine, University Hospitals Birmingham NHS Foundation Trust, Birmingham, United Kingdom; h Department of Allergy, Immunology and Rhinology, Royal National ENT Hospital, London, United Kingdom; i Division of Immunity and Infection, University College, London, United Kingdom; j Department of Immunology and Immunotherapy, School of Infection, Inflammation and Immunology, University of Birmingham, Birmingham, United Kingdom; k Department of Allergy and Immunology, University Hospitals Birmingham NHS Foundation Trust, Birmingham, United Kingdom

**Keywords:** Allergic rhinitis, phenotypes, cluster analysis, house dust mite, India

## Abstract

**Background:**

Allergic rhinitis (AR) affects approximately 10% of Indian adults, but its clinical and biological heterogeneity remains poorly defined.

**Objective:**

We sought to characterize AR phenotypes in South Indian adults using clinical features, spirometry, fractional exhaled nitric oxide (Feno), skin prick tests, blood biomarkers, and cluster analysis.

**Methods:**

This was a prospective observational study of 122 patients (≥18 years old) with AR with or without asthma attending a South Indian tertiary allergy clinic and 50 asymptomatic nonatopic control subjects. Participants underwent standardized symptom/exposure assessments, spirometry, Feno, blood counts, serum IgE, and skin prick tests to 10 aeroallergens. Principal component analysis, correlation networks, and unsupervised k-means clustering were applied to define phenotypes.

**Results:**

Patients with AR were younger than control subjects, predominantly female, and frequently exposed to incense and mosquito repellents. Sensitization to house dust mite (>70%) and polysensitization were common. Compared with control subjects, patients with AR had elevated Feno (43 vs18 ppb; *P* < .01) but no significant differences in serum IgE or eosinophils. Spirometry revealed modestly lower FEV_1_/forced vital capacity in patients with AR (*P* < .01), though values remained within normal limits. Cluster analysis identified 3 subgroups: high Feno, low eosinophils, moderate-to-severe AR with less asthma; low Feno, high eosinophils, with more asthma; and impaired lung function with moderate eosinophilia and the highest asthma burden. Network analysis demonstrated strong cosensitization between house dust mite, *Parthenium hysterophorus* (weed pollen), *Cynodon dactylon* (Bermuda pollen), and cockroach.

**Conclusions:**

This study showed 3 distinct AR clusters with high dust mite sensitization alongside cosensitization with cockroach, weed, and Bermuda pollens. Multicenter studies are warranted to further refine AR clusters, including tissue-level biomarker profiling and relevance to pharmacotherapy and immunomodulatory therapies.

## Introduction

The prevalence of allergic rhinitis (AR) among Indian adults has been estimated at 9.8% (95% CI 9.55%-9.96%) based on the Global Asthma Network Phase I study,^1^ but its clinical, immunologic, and physiologic heterogeneity remains poorly defined. Phenotyping and endotyping strategies developed in high-income countries have limited validation in tropical low- and middle-income countries such as India, where environmental, genetic, epigenetic, and human behavioral factors differ.^2^ This study aimed to comprehensively characterize adults with AR in a South Indian tertiary center in Mysuru, using clinical features, spirometry, fractional exhaled nitric oxide (Feno), skin prick tests (SPTs), peripheral blood biomarkers, and unsupervised cluster analysis.

We conducted a prospective observational study including 122 sequential adult (≥18 years) patients with AR with or without asthma, recruited via the Asthma & Allergy Clinic in JSS Hospital, Mysuru. Patients on immune-modifying treatments, including recent corticosteroid/immunosuppression/biologic use or previous or ongoing allergen-specific immunotherapy, or with active infections, immunodeficiency, pregnancy, active systemic autoimmune disease, or inability to consent were excluded. A comparator group of 50 asymptomatic nonatopic individuals (based on clinical history and negative SPTs [SPTs to an aeroallergen panel, described in the next paragraph]) was also included. Control subjects were community participants drawn from the long-standing MUDHRA^3^ and BOLD^4^ cohorts in Mysuru and were included only if they were completely asymptomatic for allergic diseases and had negative SPTs to all aeroallergens. This study was approved by the Institutional Ethics Committee of JSS Medical College, Mysuru, India (JSSMC/IEC/18.02.2022/11NCT/2021-22), and University of Birmingham, Birmingham, United Kingdom (ERN_21-0069). Written informed consent was obtained from all participants.

All participants completed detailed symptom and exposure histories on a standardized study proforma that included demographics and indoor pollutant exposure, as well as frequency of incense stick or mosquito repellent use and pet ownership. AR and asthma diagnoses were based on AR and its impact according to Allergic Rhinitis and its Impact on Asthma^5^ and Global Initiative for Asthma^6^ guidelines. SPTs were performed using a panel of 10 common aeroallergens,^7^ including mixed *Dermatophagoides pteronyssinus* and *Dermatophagoides farinae*, *Canis familiaris*, *Periplaneta americana*, *Parthenium hysterophorus*, *Cynodon dactylon*, *Amaranthus spinosus*, *Artemisia vulgaris*, and *Sorghum vulgare*. A complete hemogram, serum total IgE, Feno (NIOX VERO; NIOX Group plc, Oxford, United Kingdom),^8^ and spirometry pre- and post-bronchodilator (ndd EasyOne Spirometer; ndd Medical Technologies, Zurich, Switzerland)^9^ were conducted.

Principal component analysis reduced data dimensionality and visualized interrelationships among clinical and laboratory parameters. Correlation network analysis assessed interdependencies, and unsupervised k-means clustering identified discrete patient subgroups from integrated features.

All data generated or analyzed during this study are included in this article. Data are available from the corresponding author on reasonable request.

## Results and discussion

Patients were significantly younger and had higher Feno levels (median 43.0 ppb vs 18.0 ppb; *P* < .01), indicating type 2 airway inflammation (Table I). No significant difference was found in serum total IgE levels. Absolute eosinophil counts (AECs) were significantly higher in control subjects. Both groups showed relatively low FEV_1_ and forced vital capacity (FVC), with no significant differences. FEV_1_ was modestly lower in patients, and both groups showed mild reversibility after bronchodilator. The FEV_1_/FVC ratio (pre/post-bronchodilator) was lower in patients (*P* < .01), though median values in both groups remained >80%, suggesting limited clinical impact. The slightly lower FEV_1_/FVC ratios observed in asymptomatic nonatopic control subjects likely reflect background airway variability in the general population, which is increasingly recognized in low- and middle-income countries with high exposure to traffic emissions and biomass smoke. Such environmental influences, even in the absence of clinical symptoms or sensitization, may contribute to subtle airflow limitation and have been previously observed in our community-based MUDHRA^3^ and BOLD^4^ cohorts. In our cohort, total IgE levels were broadly comparable between patients and control subjects, with overlapping distributions, whereas AECs were marginally higher in control subjects. This pattern reflects the wide physiologic variability of these markers in tropical populations, where nonatopic environmental stimuli, including pollution, infections, and other immune exposures, can elevate IgE or eosinophils even in asymptomatic, nonsensitized individuals. Similar high background IgE levels in clinically healthy South Indian subjects were previously reported in the EuroPrevall study.^10^^,^^11^

**Table I:** Baseline characteristics of patients and control subjects

	Patients (n = 122)	Control subjects (n = 50)	*P* value
Age (years), median (IQR)	35.5 (28.0-49.0)	48.0 (35.9-59.1)	< .01∗
Sex, no. (%)			.02†
Female	81.0 (66.4)	24.0 (48.0)	
Male	41.0 (33.6)	26.0 (52.0)	
BMI (kg/m^2^), median (IQR)	25.2 (22.3-27.9)	26.8 (24.0-29.4)	.07∗
State residential area, no. (%)			.69†
Rural	19.0 (15.6)	7.0 (14.0)	
Semiurban	44.0 (36.1)	18.0 (36.0)	
Urban	59.0 (48.4)	25.0 (50.0)	
Animal exposure, no. (%)			< .410†
Cattle	4.0 (3.3)	4.0 (8.0)	
Pets	18.0 (14.8)	7.0 (14.0)	
None	100.0 (82.0)	39.0 (78.0)	
Mosquito repellent, no. (%)			.816†
Liquidator	20.0 (16.4)	9.0 (18.0)	
Mosquito mat	7.0 (5.7)	4.0 (8.0)	
No	95.0 (77.9)	37.0 (74.0)	
Burning incense sticks/dhoop at home, no. (%)			.722†
No	67.0 (54.9)	23.0 (46.0)	
Rare	4.0 (3.3)	2.0 (4.0)	
Incense sticks	43.0 (35.2)	22.0 (44.0)	
Dhoop	8.0 (6.6)	3.0 (6.0)	
Total IgE, median (IQR)	312.0 (123.2-699.0)	221.0 (112.5-738.5)	.42∗
*Dermatophagoides pteronyssinus* (serum specific IgE), median (IQR)	0.7 (0.1-17.1)	0.3 (0.0-3.0)	.12∗
*Dermatophagoides farinae* (serum specific IgE), median (IQR)	0.6 (0.1-13.1)	0.2 (0.1-6.1)	.41∗
Hemoglobin (g/dL), median (IQR)	12.0 (11.0-14.5)	12.4 (11.3-13.1)	.90∗
Total count (cells/cm^3^), median (IQR)	6900.0 (6700.0-7200.0)	7570.0 (6797.5-9280.0)	< .01∗
Neutrophil (%), median (IQR)	69.0 (64.0-71.0)	62.4 (54.5-68.1)	< .01∗
Lymphocyte (%), median (IQR)	26.5 (23.0-31.0)	31.2 (25.9-38.5)	< .01∗
Monocyte (%), median (IQR)	2.0 (2.0-3.0)	2.7 (2.2-3.6)	.09∗
Eosinophil (%), median (IQR)	3.0 (2.9-4.0)	3.1 (1.9-4.0)	.09∗
ESR (mm/h), median (IQR)	25.0 (10.0-45.0)	20.0 (15.3-22.7)	.06∗
PCV (%), median (IQR)	36.8 (34.8-44.8)	36.4 (34.2-40.0)	.03∗
AEC (cells/cm^3^), median (IQR)	260.0 (196.7-360.0)	300.0 (250.0-352.5)	< .01∗
RBC count (cells × 10^12^/L)	4.6 (4.4-5.0)	4.4 (4.1-4.8)	.03∗
Platelet count (cells × 10^9^/L)	360 (280-460)	280 (250-310)	< .01∗
PLR, median (IQR)	189.7 (142.9-262.3)	116.3 (93.1-150.4)	.01∗
NLR, median (IQR)	2.4 (2.0-3.0)	2.0 (1.4-2.6)	< .01∗
Feno (ppb), median (IQR)	43.0 (19.0-59.0)	18.0 (12.0-24.0)	< .01∗
FVC predicted %, median (IQR)	71.5 (60.0-87.0)	71.5 (60.9-84.1)	.97∗
FEV_1_ predicted %, median (IQR)	69.0 (59.9-82.0)	74.5 (63.8-84.0)	.31∗
FVC post %, median (IQR)	78.0 (66.0-87.0)	74.5 (65.7-85.1)	.41∗
FEV_1_ post %, median (IQR)	78.0 (66.9-88.0)	80.0 (66.7-85.3)	.93∗
Pre FEV_1_/FVC ratio, median (IQR)	83.2 (75.2-90.6)	87.2 (83.3-96.4)	< .01∗
Post FEV_1_/FVC ratio, median (IQR)	86.0 (79.6-92.7)	90.6 (85.4-95.3)	< .01∗

*BMI*, Body mass index; *ESR*, erythrocyte sedimentation rate; *IQR*, interquartile range; *NLR*, neutrophil-to-lymphocyte ratio; *PCV*, packed cell volume; *PLR*, platelet-to-lymphocyte ratio; *RBC*, red blood cell.

∗ Wilcoxon.

† Pearson.

The patients with AR were predominantly female (66.4%) with a median age of 35.5 years (Table I). Nearly 50% resided in urban areas, and incense burning (45%) and mosquito repellent use (22%) were common exposures. Moderate-to-severe persistent AR was the dominant pattern (approximately 60%), and 65% had coexisting asthma. Greater than 70% were sensitized to house dust mite (HDM), whereas cockroach, *Parthenium*, *Amaranthus*, and *Cynodon* sensitizations were also common. Among the pollen allergens assessed, *A spinosus* sensitization was observed in 63.1% of patients; *Artemisia tridentata*, in 41.8%; *Parthenium*, in 55.7%; *C dactylon*, in 41.0%; *S vulgare*, in 24.6%; *Cocos nucifera*, in 27.9%; *Prosopis juliflora*, in 34.4%; *Ricinus communis*, in 53.3%; and *Cassia siamea*, in 26.2%. No subject reported aspirin/nonsteroidal anti-inflammatory drug sensitivity.

**Fig 1:**
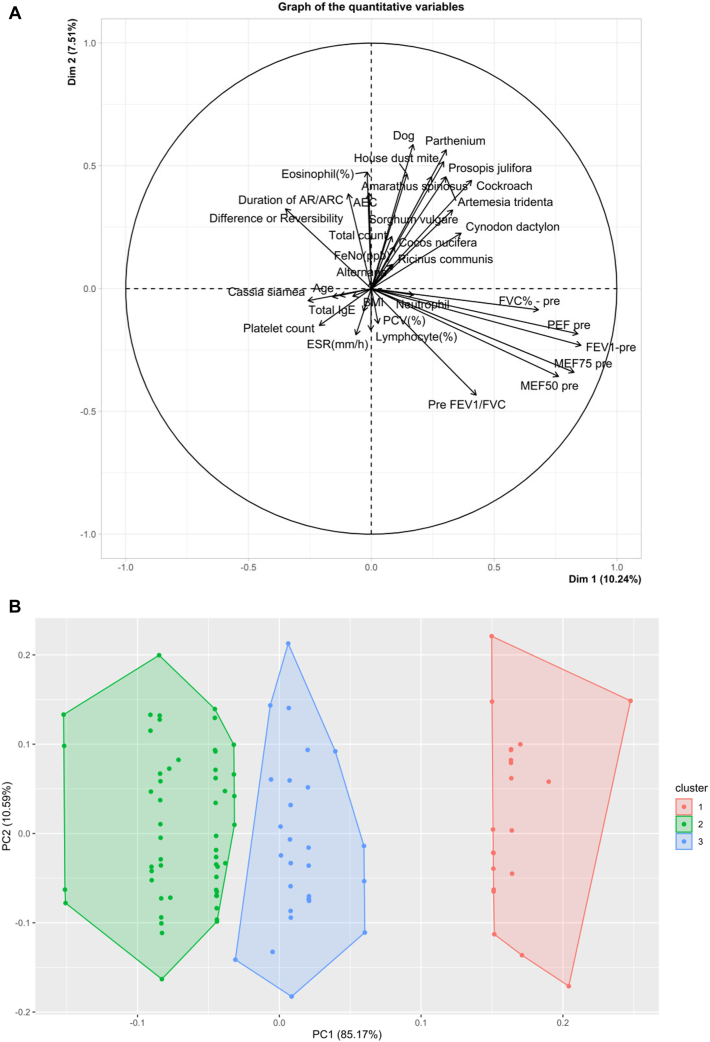
Principal component analysis biplot of quantitative variables **(A)** and k-means cluster plot of participants with AR based on multidimensional variables **(B)**. *(A)* This biplot displays the distribution and relationships of quantitative clinical, physiologic, and immunologic variables among all study participants using principal component analysis. Each *arrow* represents a variable; the direction and length indicate its contribution to variance within the dataset. The relative positioning of participant data points reflects similarities and differences across phenotypic profiles, highlighting the multidimensional spread of AR and control groups. *(B)* This scatterplot visualizes the 3 clusters identified among adults with AR by unsupervised k-means clustering, projected onto the 2 leading principal components. Each *colored point* represents an individual classified within 1 of the clusters, demonstrating the phenotypic heterogeneity in the cohort. Cluster differentiation is based on integrated profiles of Feno, eosinophils, lung function, and sensitization patterns, underscoring biologically distinct AR subgroups that emerged from multidimensional analysis. PC1 (85.01%) represents the principal axis capturing the largest proportion of variance across clinical, spirometric, inflammatory, and sensitization variables, and PC2 (10.57%) captures the next major source of variability. Together, these components allow visual separation of the 3 data-driven clusters in the plot. *ARC*, Allergic rhinoconjunctivitis; *BMI*, body mass index; *ESR*, erythrocyte sedimentation rate; *MEF50/75*, maximal expiratory flow at 50%/75%; *PEF*, peak expiratory flow.

Three AR clusters were identified, distinguished by integrated immunologic and physiologic profiles (Table II, Fig 1). Cluster 1 had high Feno (77% with >25 ppb), low eosinophils (mean AEC 161.9 cells/μL), and moderate-to-severe AR with less asthma; sensitized to multiple allergens, especially HDM (mixture of *D pteronyssinus* and *D farinae*), this cluster had the highest percentage of subjects with moderate-to-severe AR (63.9%) and the lowest percentage of subjects with asthma (52.5%). Cluster 2 had low Feno (45.5% with >25 ppb), high eosinophils (mean AEC 523.2 cells/μL), and polysensitization; the proportion of subjects with moderate-to-severe AR (59.1%) was lower than in cluster 1, and the proportion of subjects with asthma (68.2%) was higher than in cluster 1. Cluster 3 had the most impaired lung function (FEV_1_/FVC 75.2%), lower FEV_1_% predicted (68% predicted), lower pre–peak expiratory flow and maximal expiratory flow at 50%/75%, and moderate eosinophilia (mean AEC 292.7 cells/μL); this cluster had the least number of subjects with moderate-to-severe AR (54%), but the highest asthma burden (73%) and elevated Feno (70.3% with >25 ppb).

**Table II:** Baseline characteristics of study participants by cluster analysis

	Cluster 1 (n = 62)	Cluster 2 (n = 22)	Cluster 3 (n = 38)	*P* value
Religion (%)				.1991
Christian	8.2	9.1	2.7	
Hindu	80.3	77.3	94.6	
Muslim	11.5	13.6	2.7	
Age (years), mean ± SD	40.3 ± 15.2	39.7 ± 12.9	35.1 ± 12.1	.2104
Age (%)				.5204
<20	4.9	0	5.4	
20-40	50.8	59.1	64.9	
>40	44.3	40.9	29.7	
Sex (%)				.5013
Male	36.1	40.9	27.0	
Female	63.9	59.1	73.0	
AR severity				.6231
Moderate-to-severe intermittent AR (%)	36.1	40.9	46.0	
Moderate-to-severe persistent AR (%)	63.9	59.1	54.0	
Asthma (%)	52.5	68.2	73.0	.1011
Duration of AR or rhinoconjunctivitis, mean ± SD	7.9 ± 9.5	11.8 ± 12.5	9.5 ± 9.2	.4346
BMI (kg/m^2^), mean ± SD	26.6 ± 7.4	26.1 ± 4.6	24.3 ± 4.0	.3405
BMI (%)				.6105
<25	50.8	40.9	54.0	
≥25	49.2	59.1	46.0	
HDM (%)				.2064
Wheal size ≤3 mm	14.8	9.1	2.7	
Wheal size 3-6 mm	32.8	36.4	24.3	
Wheal size >6 mm	52.4	54.5	73.0	
Cockroach (%)				.4624
Wheal size ≤3 mm	44.3	31.8	32.4	
Wheal size 3-6 mm	50.8	68.2	64.9	
Wheal size >6 mm	4.9	0	2.7	
Dog (%)				.0748
Wheal size ≤3 mm	72.1	45.4	67.6	
Wheal size 3-6 mm	27.9	54.6	32.4	
*Amaranthus spinosus* (%)				.1320
Wheal size ≤3 mm	36.1	45.5	35.1	
Wheal size 3-6 mm	57.4	31.8	56.8	
Wheal size >6 mm	6.5	22.7	8.1	
*Artemisia tridentata* (%)				.0147∗
Wheal size ≤3 mm	59.0	31.8	70.3	
Wheal size 3-6 mm	29.5	45.5	29.7	
Wheal size >6 mm	11.5	22.7	0	
*Parthenium hysterophorus* (%)				.8032
Wheal size ≤3 mm	41.0	36.4	51.4	
Wheal size 3-6 mm	50.8	54.5	43.2	
Wheal size >6 mm	8.2	9.1	5.4	
*Cynodon dactylon* (%)				.2489
Wheal size ≤3 mm	57.4	50.0	67.6	
Wheal size 3-6 mm	36.1	36.4	32.40	
Wheal size >6 mm	6.5	13.6	0	
*Sorghum vulgare* (%)				.8440
Wheal size ≤3 mm	91.8	95.4	91.9	
Wheal size 3-6 mm	8.2	4.6	8.1	
Eosinophil (%), mean ± SD	2.6 ± 0.9	7.0 ± 1.3	4.4 ± 1.3	< .0001∗
AEC, mean ± SD	161.9 ± 46.1	523.2 ± 34.2	292.7 ± 32.2	< .0001∗
FVC predicted %, mean ± SD	71.4 ± 18.8	71.5 ± 15.9	74.6 ± 16.1	.7684
FEV_1_ predicted %, mean ± SD	71.4 ± 18.3	71.5 ± 17.0	68.0 ± 20.0	.2811
Pre FEV_1_/FVC ratio, mean ± SD	83.8 ± 15.4	81.2 ± 14.2	75.2 ± 16.8	.0143∗
Pre PEF, mean ± SD	77.9 ± 21.9	77.3 ± 19.3	66.2 ± 20.8	.0082∗
Pre MEF75, mean ± SD	77.1 ± 25.7	76.8 ± 30.7	61.5 ± 30.2	.0292∗
Pre MEF50, mean ± SD	67.8 ± 29.6	70.1 ± 36.0	50.5 ± 27.9	.0155∗
Difference or reversibility, mean ± SD	6.9 ± 8.6	8.0 ± 7.7	7.7 ± 8.0	.6671
Feno (%)				.0158∗
<25 ppb	22.9	54.5	29.7	
25-50 ppb	39.4	9.1	46.0	
>50 ppb	37.7	36.4	24.3	
Total count (cells/mm^3^), mean ± SD	6843 ± 1291	7254 ± 306	6976 ± 416	.0022∗
Neutrophil %, mean ± SD	66.0 ± 13.1	66.6 ± 10.5	66.3 ± 3.5	.0835
Lymphocyte %, mean ± SD	27.4 ± 5.9	23.2 ± 5.2	28.8 ± 3.6	.0003∗
Monocyte %, mean ± SD	0.95 ± 1.2	1.5 ± 2.1	0.9 ± 1.2	.4168
ESR (mm/h), mean ± SD	37.8 ± 27.6	21.5 ± 16.0	27.7 ± 17.0	.0648
Platelet count (cells × 10^9^/L), mean ± SD	400 ± 160	330 ± 95	370 ± 93	.1595
PLR, mean ± SD	216.0 ± 91.6	209.6 ± 87.9	186.0 ± 53.8	.3390
NLR, mean ± SD	2.6 ± 0.93	3.0 ± 0.50	2.3 ± 0.41	.0010∗

*BMI*, Body mass index; *ESR*, erythrocyte sedimentation rate; *MEF50/75*, maximal expiratory flow at 50%/75%; *NLR*, neutrophil-to-lymphocyte ratio; *PEF*, peak expiratory flow; *PLR*, platelet-to-lymphocyte ratio.

∗ Statistically significant value.

**Fig 2:**
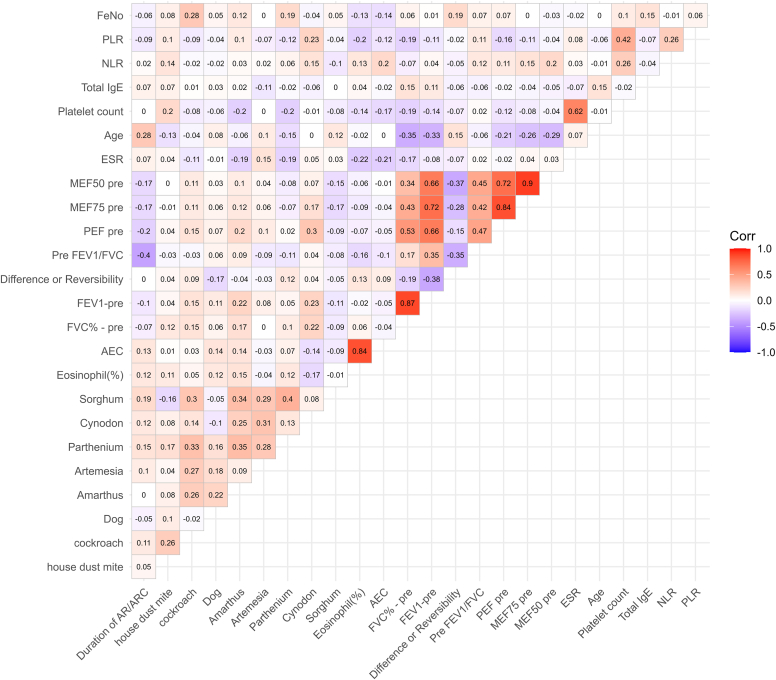
Correlation heatmap of clinical, inflammatory, spirometric, and sensitization variables in study participants. *Red* represents strong positive correlations; *blue* indicates strong negative correlations. *ARC*, Allergic rhinoconjunctivitis; *ESR*, erythrocyte sedimentation rate; *MEF50/75*, maximal expiratory flow at 50%/75%; *NLR*, neutrophil-to-lymphocyte ratio; *PEF*, peak expiratory flow; *PLR*, platelet-to-lymphocyte ratio.

A comprehensive visualization of interrelationships among clinical, inflammatory, spirometry, and sensitization variables is shown in Fig 2. The correlation heatmap demonstrated strong within-domains correlations, particularly among spirometry indices (FEV_1_, FVC), inflammatory markers (eosinophils, Feno), and allergen sensitization parameters. Cross-domain links were evident, with Feno and AEC strongly linked to aeroallergen sensitization, reflecting the inflammatory and immunologic complexity of AR. Notably, total IgE correlated with sensitization, but not with Feno or lung function. These findings underscore the multidimensional and interconnected nature of AR in this cohort, highlighting a pattern of polysensitization and systemic inflammation rather than isolated single-marker profiles.

**Fig 3:**
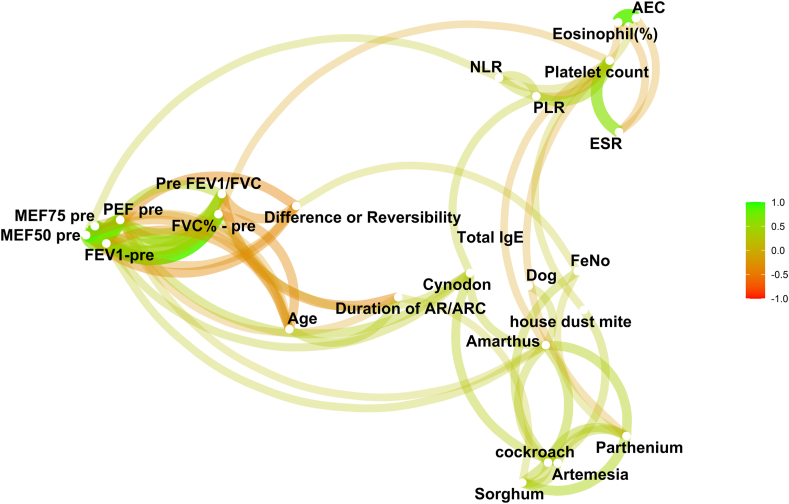
Network graph that depicts a comprehensive visual synthesis of pairwise correlations among clinical, immunologic, and physiologic variables in individuals with AR. This network diagram visualizes the complex interrelationships among aeroallergen sensitizations, blood eosinophil counts, and lung function measures in the AR cohort. Each node represents a specific variable, such as allergens, eosinophils, and lung function indices. Edges *(lines)* between nodes indicate statistically significant associations, with proximity reflecting the strength of these relationships: nodes closer together have stronger coassociations. The color gradient of the edges ranges from *green* to *red*, where *green lines* represent positive associations, and *red lines* represent negative associations. The network reveals clusters of cosensitized allergens alongside linked inflammatory and functional measures, providing a holistic view of the multidimensional interactions underlying AR phenotypes. *ARC*, Allergic rhinoconjunctivitis; *ESR*, erythrocyte sedimentation rate; *MEF50/75*, maximal expiratory flow at 50%/75%; *NLR*, neutrophil-to-lymphocyte ratio; *PEF*, peak expiratory flow; *PLR*, platelet-to-lymphocyte ratio.

Network analysis revealed highly interconnected sensitization patterns, with strong cosensitization links among *P hysterophorus*, *C dactylon*, HDM (*D pteronyssinus*), and cockroach, suggesting shared exposure pathways. Importantly, the network structure emphasized a polysensitized phenotype rather than isolated single-allergen sensitization, supporting the observation that allergen-driven inflammation in this Indian cohort differs from European monoallergen-dominated patterns (Fig 3). Feno was directly related to allergen sensitization and to AEC, but not to lung function. AEC correlated with sensitization, but not with lung function. Total IgE was strongly linked to allergen sensitization only.

AR studies in India have concentrated on prevalence surveys and sensitization patterns, with limited attention to integrative, multidimensional phenotyping. Our work moves the field forward by simultaneously incorporating clinical features, spirometry, AEC, and serum total IgE, and allergen sensitization profiles, enabling the delineation of distinct biological subgroups within AR.

The high prevalence of polysensitization dominated by HDM (*D pteronyssinus* and *D farinae*) and *P hysterophorus* is consistent with previous Indian studies highlighting the complex allergen landscape in tropical and urban environments.^12^^,^^13^ Similar polysensitization patterns have been reported in European^14^, ^15^, ^16^, ^17^ and East Asian^18^ populations, underscoring that multiple allergen exposures are increasingly the rule rather than the exception in adult AR. Our findings reflect a convergence with global trends, although the dominance of *Parthenium* highlights important geographic differences.

Cluster 1 aligns with the type 2 airway inflammation without systemic eosinophilia phenotype described in European^19^ and Chinese^20^ cohorts. Cluster 2 corresponds to the eosinophil–high systemic inflammation phenotype noted in large multicenter analyses such as the Global Allergy and Asthma European Network.^21^ Cluster 3 is similar to lower airway–dominant AR phenotypes described in both Western and East Asian settings.^15^^,^^19^^,^^22^, ^23^ These alignments with these international archetypes suggest that despite differences in allergen exposure and environmental context, the biological underpinnings of AR heterogeneity demonstrate broad global consistency.

Notably, we identified a subset of asymptomatic, nonsensitized control subjects with unexpectedly elevated AEC and total IgE levels. This subclinical immunologic activation has been observed in other tropical cohorts,^24^, ^25^ where high background exposure to ambient air pollution, biomass smoke, helminths, or viral infections can amplify type 2 immune responses independently of clinical allergy. Such findings emphasize the need to interpret serum total IgE and AEC cautiously in high-exposure environments, where they may not map neatly onto clinical disease.

In tropical settings, chronic or past helminth exposure is well recognized to elevate total IgE and peripheral eosinophil counts independent of aeroallergen sensitization, thereby complicating the interpretation of systemic atopy markers. Although active parasitic infections were not evaluated in our cohort, this background immunologic influence remains an important contextual factor in South Asia and may partly account for the heterogeneity in IgE and eosinophil levels observed across clusters.

Our results reinforce that AR cannot be adequately defined by clinical severity scales or sensitization patterns alone. Integrative clustering, combining clinical, physiologic, and systemic inflammatory data, provides a more nuanced understanding of disease heterogeneity. Although our study was not designed to test treatment response or prognosis, the identification of distinct AR clusters provides a foundation for future longitudinal and interventional studies in India and possibly other tropical low- and middle-income countries.

A key strength of this study is the multidimensional characterization of adult AR using accessible clinical assessments, immunologic profiling, and a data-driven clustering approach. We excluded important confounders such as active infections, systemic autoimmune disorders, immunodeficiency, as well as confounders of immunosuppression and/or immunomodulatory therapies. The inclusion of a well-defined community control group enabled meaningful comparisons and strengthened internal validity. Network analysis provided novel insights into cosensitization patterns between *P hysterophorus*, *C dactylon*, HDM (*D pteronyssinus*), and cockroach. Limitations include a single-center design, a modest sample size that limits generalizability, and a lack of stool examination to exclude individuals with helminthic infestation. The study population is relatively homogeneous, and therefore findings may not be generalizable to other geographic or ethnic groups. In the EuroPrevall study in the same population, stool PCR was evaluated for helminth infestation, and it showed 11% positivity for helminths (J. J. Janse, MSc, unpublished data, May 2014). However, in this study, subclinical infections could contribute to variations in eosinophil counts and total IgE levels, and helminthic infestation has not been evaluated in this study. The absence of longitudinal follow-up prevents assessment of the temporal stability of clusters or their predictive value for clinical outcomes. Network analysis, although informative, was exploratory and not validated in an external cohort.

Our data identified 3 AR clusters in this South Indian cohort. Our results support the use of cluster-based approaches in AR to capture biological heterogeneity that may not be evident from standard clinical labels alone. Although our study was not designed to evaluate outcomes, the identification of biologically distinct AR phenotypes underscores that patients classified under the same clinical label may have differing inflammatory profiles and airway involvement. Recognizing these subgroups may help refine clinical assessment and guide more targeted management approaches in similar tropical settings and needs further study. Future work should validate these clusters in larger, longitudinal multicenter cohorts and explore their clinical relevance.

## Disclosure statement

This research was funded by the Global Challenge Research Fund secured via the 10.13039/501100000855University of Birmingham, Birmingham, United Kingdom. M.K.U. is a recipient of the JSS Academy of Higher Education & Research Post Doctoral Fellowship (Order No. JSS AHER/REG/RES/JSS AHER PDF/720/2024-25) and the Senior Research Fellowship award (Fellowship Sanction No. 45/13/2022/TRM/BMS) from the 10.13039/501100001411Indian Council of Medical Research.

Disclosure of potential conflict of interest: The authors declare that they have no relevant conflicts of interest.
